# Conservative Management of Acute Lateral Ligaments of the Ankle Injuries: An Analytical Literature Review

**DOI:** 10.7759/cureus.47709

**Published:** 2023-10-26

**Authors:** Godsfavour C Maduka, Ruta Jakusonoka, Divinegrace C Maduka, Naeem Yusuf

**Affiliations:** 1 Trauma and Orthopaedics, Lister Hospital, East & North Herts National Health Service (NHS) Trust, Stevenage, GBR; 2 Orthopaedics, Riga Stradins University, Riga, LVA; 3 Major Trauma, Queens Medical Centre-Nottingham University Hospitals National Health Service (NHS) Trust, Nottingham, GBR; 4 Plastic Surgery, Lister Hospital, East & North Herts National Health Service (NHS) Trust, Stevenage, GBR

**Keywords:** acute ankle sprain, return to activity, functional management, conservative management, ptfl, cfl, atfl, lateral ligaments of ankle

## Abstract

Injuries to the lateral ligaments of the ankle are among the most frequent sporting injuries. These injuries constitute a significant portion of all sports-related injuries. Nearly all cases involve damage to either the anterior talofibular ligament (ATFL) or the calcaneofibular ligament (CFL). While they are generally considered to be mild injuries, without adequate rehabilitation and treatment, these injuries often result in lingering symptoms for many patients for a period ranging from 6 weeks to 18 months.

Subsequently, this analysis seeks to assess the non-surgical, conservative approaches currently employed in managing lateral ligament injuries of the ankle. Therefore, this assessment explores the variations and effectiveness of conservative treatment approaches based on the injury's severity and the mechanisms of trauma.

The study conducted an analytical literature review that relied on diverse sources, including orthopedic books, e-books, articles, journals, and internet databases, to accomplish this. The main sources were obtained from reputable databases such as UpToDate, NCBI, and PubMed. Collectively, these sources provide definitions, outlines, evaluations, and discussions related to the topic. As such, they facilitated the formulation of an informed conclusion on the approach to treating lateral ligament injuries of the ankle complex.

The reviewed literature shows that early and effective initial treatment involving pain management, prompt resumption of weight-bearing activities, limited immobilisation, and targeted physical therapy yields favorable outcomes for minor-grade sprains and is an effective preventive measure against recurrent injuries. Accordingly, athletes who experience regular ankle sprains should consider prophylactic bracing or taping to lower the risk of re-injury while enhancing their functionality.

Notably, the existing functional and conservative management methods demonstrate and yield positive post-treatment outcomes. Nonetheless, the efficiency and effectiveness of these treatments depend on the specific nature of the injury and the unique traits of the individuals who sustain it. Consequently, these factors must be considered for when determining the appropriate treatment approach.

## Introduction and background

Intrasubstance injuries to the ligaments of the ankle, such as sprains or tears, are documented as some of the most prevalent sports-related injuries, particularly in sports involving high-energy activities such as jumping, running, and rapid changes of direction [1]. These injuries can occur in everyday activities and are not exclusive to sports, gender, or age-specific since one can incur a sprain from stepping on uneven surfaces or descending at an awkward angle [2].

Statistically, there is a considerable incidence of lateral ligament ankle injuries, with the United States accounting for approximately 23,000 cases and the United Kingdom for 5,600 daily cases [3-4]. This high occurrence is coupled with high chances of enduring post-traumatic symptoms such as instability and chronic pain [3]. This underscores the need for a standardized, evidence-based treatment methodology, informed by current literature, to enhance positive and optimal outcomes following management.

Ankle injuries account for a significant portion (25%) of all sporting injuries, with about 20% in soccer and 21% in basketball [4-6]. Ankle ligaments can be categorized into two major groups, lateral and medial, which collectively form the ankle joint. The lateral collateral ligament of the ankle is comprised of the posterior talofibular ligament (PTFL), the calcaneofibular ligament (CFL), and the anterior talofibular ligament (ATFL), arranged in a fan-like pattern [2]. The lateral ligaments are the most affected in intrasubstance ankle sprains since they are designed to deter inward rolling and prevent excessive ankle inversion, subjecting them to about 80% of ankle damage [1].

Consequently, this primary mechanism of injury affects the ATFL (65% of cases), although severe inversion or traumatic force can result in complete rupture, affecting the CFL and PTFL [1]. Accordingly, these injuries constitute a substantial portion (approximately 25%) of all sports-related injuries, of which over 90% are attributed to CFL or ATFL damage [4].

Ankle sprains are graded based on severity, ranging from grade 1 to grade 3, which guides the approach to prognosis, rehabilitation, and treatment. Despite ankle sprains being relatively mild, ineffective rehabilitation and treatment often result in lingering symptoms for about 55% to 72% of patients for a period ranging from 6 weeks to 18 months [7].

These injuries can be managed with surgical intervention or conservative methods with functional management or immobilisation [8]. Essentially, crucial parameters for assessing the effectiveness of treatment regimens focus on the rates of re-injury or recurrence, the duration of disability during recovery and post-treatment rehabilitation, chronic ankle pain, the incidence of post-traumatic lateral ankle instability, and functional outcomes post-treatment [2,4].

The analysis seeks to assess the conservative methods currently employed in managing ankle injuries to the lateral collateral ligament, as explained in published literature. Ideally, the evaluation seeks to expose the effectiveness of these conservative methods and how their variations in application are influenced by the severity of the injury and the mechanisms of trauma.

## Review

Method

The study involved a comprehensive analytical literature review following generally accepted international principles. This review included sources from UpToDate, NCBI, PubMed, NCBI, and other internet databases to access e-books, articles, journals, and other orthopaedic publications. The sources selected for review provided definitions, outlines, evaluations, and discussions related to ankle injuries, especially lateral ligament damage. As such, the approach allowed for a thorough evaluation to establish conclusions related to the treatment methods utilized for managing injuries to the lateral ligaments of the ankle complex.

The analyses, results, and non-experimental information presented in this study are drawn from accessible studies published in the 20th and 21st centuries. These sources include peer-reviewed scientific studies published in reputable database registries, as well as content sourced from clinical guidelines and textbooks from reputable governing bodies. These resources are accessible to qualified professionals and students in the field.

Criteria for Inclusion or Exclusion of a Study

Studies with irrelevant, inaccessible, contradictory, or incomplete information were excluded from the analysis. Racial and gender differences among study subjects were inconsiderable during the selection of the studies for inclusion. In addition, the authors' nationality, origin, and origin of their publications were irrelevant and not included as a parameter for the inclusion or exclusion of studies. As such, the main criteria adhered to were that the study had undergone peer review and was published in databases or journals accessible for data collection [9]. A list of the referenced studies and articles is included in the bibliography.

For inclusion in this review, the articles and journals must have focused on investigating patients between 12 and 65 years old without any significant pre-existing congenital deformities or chronic comorbidities, such as congenital bone deformities, diabetes, or cancer. Accordingly, the criteria included all quasi-randomized and randomized controlled clinical trials that compared or evaluated approved conservative lateral ligament injuries of the ankle management methods. In addition to these criteria, particular attention was given to more recent information-studies between 2010 and 2023-to ensure the most contemporary findings were considered in the analysis. Due to the heterogeneity of the topic and the limited number of high-quality studies available in the literature; studies completed no earlier than 1993 were also included in the review. This allows for comparison and identification of changing trends and methods of management while maintaining an adequate number of studies for review. 

Data Collation and Organisation

The qualifying 38 sources out of 60 initially identified sources were grouped by similarity and comparability by factors such as age of participants, type/grading method of injuries assessed, management plan, or treatment modality. Since the racial and gender specifications of participants were not relevant for this study, no selection or grouping was carried out based on these factors. As such, the 38 sources were organized into groups where data extraction and profiling were conducted based on the grading of injuries, participant age, types of injuries, and the management and treatment approach applied. This systematic categorization facilitated the analysis of the data and the generation of results [10-11].

Data Analysis

The analysis involved an assessment of the quality and risk of bias in the included randomized controlled trials (RCTs) based on the Cochrane Risk of Bias Tool [12]. In addition, the quality of the included observational studies was evaluated using the Newcastle-Ottawa Scale [13]. To establish the quality of evidence for each outcome, the study applied the Grading of Recommendations Assessment, Development, and Evaluation (GRADE) approach [14-16].

A comparative analysis was employed following the selection, compilation, and organization of data gathered from clinical studies. The analysis focused on studies that employed similar or related controlled variables and experimental procedures. Subsequently, their outcomes were compared to establish the most effective treatment or management plan within each group.

Furthermore, the study compared studies that investigated different treatment methods for similar injuries. For instance, the study compared different treatment approaches for injuries of similar grades, provided that the grading system was consistent across the studies. Finally, to validate our results, the study cross-referenced the outcomes with guidelines from reputable medical boards and professional bodies, scholarly journals, and medical textbooks (eBooks) [8].

Results

Description of Conservative Management Methods

Management of ankle injuries that result in intra-substance damage to the lateral ligament revolves around three phases: the "inflammatory phase," “the proliferation phase," and “the maturation or remodelling phase," which promote ligament healing [8,17]. The most efficient approach facilitates healing at every level/phase [2,18]. The main methods of management are described in the literature: conservative and surgical management [2,8,18,19]. The scope of this review focuses on conservative management, which is mainly carried out via functional management or cast immobilisation in a below-knee cast for 4-6 weeks.

The approach to managing these lateral ligament injuries is hinged on factors such as the patient's age, which can affect suitability for surgical intervention and post-op outcomes; prior activity level, which can affect suitability of immobilisation and return to activity; previous treatment outcomes, which can guide the decision between surgical or conservative management and the likelihood of positive outcomes; ankle instability and severity of the injury, which can again determine the suitability of conservative management and the possibility of positive outcomes post-management; and the phase of the injury at present [8,19,20]. Frequently, conservative management is the go-to therapeutic approach since it can be effectively applied in diverse cases of injury [18]. Surgical intervention may be considered subsequently depending on patient conditions such as personal preference, failed conservative management, or chronic instability [21].

Conservative management with functional therapy can be used severally or in conjunction with cast immobilisation, which offers alternative management other than the more inversive surgical intervention [9]. Uniquely, guidelines for functional treatment focus on supporting the biological healing process [22,23]. Consequently, immediate care is crucial in the initial phases of treatment since focus is given to mitigating inflammation and swelling to prevent further injury [23]. It also prepares the tissues for the entire rehabilitation process and recovery [9,24].

In the aftermath of an injury, the body's tissue responds with new collagen generation processes, fibroblast proliferation, and vascular ingrowth. The authors have divided functional therapy for lateral ligament injuries of the ankle into various phases. Some categorize it into five phases: prophylactic, functional, rehabilitative, acute, and subacute [9,22,25], while others break it down into three phases: rehabilitation, immobilisation, and PRICE (Protection, Rest, Ice, Compression, Elevation) [26,27]. During the post-injury phase, especially in severe and acute cases of ankle injury, the appropriate functional therapy and rehabilitation framework must align with four essential requirements that reflect the physical therapy phases as supported by the biological basis of wound healing [2,4]. The initial phase applies the RICE model by providing rest, ice (cold application), compression (using a tubigrip or hand), and elevation [27]. Primarily, the ultimate goal is to lower inflammation, swelling, haemorrhage, pain, and cellular metabolism, which provides an optimal environment for the healing process [25]. These measures must be implemented in the initial hours or days following the injury [4].

Second, preventing further damage during the initial weeks (1-3 weeks) of ligament injury is crucial to protecting the affected ligament and promoting conservative management. Ideally, facilitating ankle support uninterruptedly allows fibroblasts to infiltrate the injured zone and foster maximum production and proliferation of collagen fibers. Premature mobilisation may result in excessive inversion, which prolongs the formation of type III collagen, that is less robust in healing the tissues compared to optimal immobilisation [26,28].

Furthermore, protecting the injury lowers the incidence of premature lengthening and stretching of the healing ligaments and deters secondary injuries in the affected area. While immobilisation may be appropriate for protecting the ligaments, studies reveal that more favourable outcomes are observed without an immobilisation boot or with a brief immobilisation period. Accordingly, limited immobilisation mitigates the adverse impact on joints and muscles that may be affected by prolonged immobilisation [23,27].

Approximately 10 to 21 days post-injury, pain and inflammation subside and mark the onset of the maturation phase, where collagen develops and covers the wound with scar tissue [24]. During this phase, the introduction of monitored and controlled mobilisation exercises is essential for the injured ligaments. Ideally, immobilisation must be removed before this period to limit adverse immobilisation on the affected area's tendons, muscles, bones, joints, and other adjacent healthy ligaments [24]. Subsequent and successive controlled muscle exercise and joint movement therapy enhance the organization of collagen fibres along the anatomical stress lines to enhance the structural and mechanical performance of the ligaments [22,24].

Phase 3 begins four to eight weeks post-injury, with the objective of rapid rehabilitation to enable a return to work and sporting activities after full recovery. In this phase, the newly formed collagens can sustain pressure and stress on the previously affected ligaments. In the early stages of this phase, conservative management focuses on restoring proprioception, which is often compromised by the injury. The modified Romberg test establishes the level of proprioceptive deficits, often exacerbated by prolonged immobilisation or non-weight bearing, which may further injure the ligament if not intervened appropriately [4,22]. In the latter part of phase 3, the athlete undergoes readaptation in readiness to return to the field or sporting activities. Ideally, the period entails transitioning from rehabilitative to specific field skills exercises such as side-stepping, pivoting, jumping, or sprinting. This is granted to the athlete when they can engage in sport-specific activities with flexibility and less pain so that they can fully return to their sports activities [6,17].

Management protocols based on the five phases may vary slightly since the timing of each phase depends on the grade and severity of the injury. For instance, the acute phase follows PRICE principles, while the subacute phase concentrates on eliminating and reducing pain, preserving protection against re-injury with bracing, expanding the pain-free and flexible motion, enhancing approaches that reduce effusion, and mitigating strength loss through isometric exercises. On the other hand, the rehabilitation phase targets initiating less painful and free movement of the joints through stretching and mobilisation, integrating proprioceptive tilt-board exercises, and building strength via isokinetic and isotonic exercises. In the functional phase, priority is given to sport-specific exercises to achieve full functionality of affected joints and enable athletes or individuals to resume active participation in their specific activities. Finally, the prophylactic phase addresses the likelihood of injury recurrence by instilling preventive approaches, including taping and braces, among other specific prophylactic support systems. In addition, the phase includes functional proprioceptive drills and strengthening exercises that support the patient in achieving resilience [17].

Consequently, different functional management approaches have been established to provide flexible ankle injury rehabilitation tailored to the needs of each individual [29]. They incorporate isotonic, isometric, and muscular strength training, proprioception of ankle joints, flexibility, and balance exercises. In addition, other customized functional therapies, such as water resistance training, suit individual patient needs [30].

Other than exercise-based therapy, auxiliary therapeutic approaches have emerged and are applied to expedite healing and recovery. These approaches include short waves, temperature-contrast baths, ultrasound, cryotherapy, NSAIDs, or advanced approaches such as interference or diadynamic current therapy [31]. Electrical muscle stimulation (EMS) or electro-galvanic stimulation may also aid in mitigating calf muscle atrophy and enhancing muscle coordination and joint range of motion. However, it is worth noting that there are limited studies that establish the efficacy of these methods.

Before granting permission to return to active participation, patients must be assessed to ensure they are fit for full sporting exercises. The patients must demonstrate the ability to run and perform high-speed maneuvers painlessly, achieve 90% strength in the affected ankle relative to the healthy side, and experience unrestricted and painless ankle range of motion. An effective rehabilitation program incorporates a well-defined plan for management, regular monitoring, and subsequent follow-ups to support patient progress and enable them to perform appropriate exercises suitable for their stage of recovery [32]. Collaborating with a qualified physical therapist or a professional athletic trainer throughout the phases of rehabilitation can enhance the efficacy of and compliance with the rehabilitation regimen. Accordingly, this can lower the likelihood of failure in the conservative management approach and eliminate the gravitation toward a surgical intervention [2,22].

Evaluation and Discussion

The current state of available evidence for guiding conservative treatment is, regrettably, suboptimal due to inherent challenges in controlling variables and achieving comparability among individuals participating in studies. Even with the high incidence of lateral collateral ligament injuries of the ankle and the substantial volume of existing literature, achieving conclusive results remains challenging.

Different studies have sought to compare the two primary methods of treatment, such as the work by Kerkhoff et al. [33], whose findings were supported by Tiling et al. [34], Jones et al. [35], and Kannus et al. [36] in their individual reviews. Across these studies, a consensus emerged: immobilisation did not yield optimal outcomes. Instead, statistically significant results were consistently observed for patients managed with functional treatment across various domains, including patient satisfaction, persistent swelling, objective instability, the time required to return to work and sports, and the number of patients returning to sport and work [33,6,21].

Notably, Kerkhoffs et al.'s systematic review, which is now considered somewhat outdated, involved 21 trials and 2184 participants. The review concluded that functional exercises surpassed immobilisation in enhancing patient satisfaction through regaining range of motion, restoring ankle stability, reducing persistent swelling, and facilitating the return to sports and work [33].

Nonetheless, Lam et al.’s [37] single-blinded randomized controlled study advocated cast immobilisation in conservative management. This study compared a below-knee cast, Bledsoe boot, and Aircast brace against a double-layer tubular compression bandage to establish their effectiveness in enhancing recovery following severe ankle sprains. Their findings suggested that a 10-day period of complete immobilisation using an Aircast® brace or in a below-knee cast was more effective in patient recovery than only relying on the tubular compression bandage. Nonetheless, the study failed to relate functional treatment to the different methods of immobilisation and had significant methodological flaws that affected the validity and reliability of the results [32,38].

A meta-analysis conducted by Lynch and Renström [39] noted that functional management resulted in swift recovery for Grade I and II injuries. Ideally, this approach incorporates the immediate implementation of the RICE protocol, a brief period of immobilisation and protection with bandage or tape, and early initiation of neuromuscular training, weight-bearing, and range of motion exercises. The meta-analysis recommends functional management as the ideal initial therapy for faster recovery and a return to normalcy with minimal complications and fewer adversity mechanical stability. It also emphasizes the need for rehabilitative approaches for enhancing ankle functionality and balance, such as proprioceptive training on a tilt board [39].

Accordingly, critical considerations involve assessing the affected ligaments' healing potential using imaging modalities and arthroscopy for visualization [40]. The decision-making process must determine whether ligament healing with adequate tension can be achieved as effectively with early controlled mobilisation as through direct visualization and suturing [19,31]. For instance, MRI visualisations reveal that early mobilisation promotes the continuity of ligamentous healing in grade III ruptures. These studies provide evidence of early mobilisation in functional therapy to enhance ankle ligament recovery [40].

The effectiveness of auxiliary therapies aside from cryotherapy and NSAIDS in providing therapeutic benefits has not been shown in randomized controlled studies [41,42]. Only these two auxiliary therapies will be further explored due to the insufficiently appropriate literature identified for the other auxiliary therapies. However, there are less robust studies on cryotherapy since it is used in combined therapy for ankle injuries. Nonetheless, cryotherapy effectively limits inflammation and swelling and reduces the need for pain relievers, especially when applied shortly after the injury. Cryotherapy is also essential in clinical examination and is part of the recommended phase 1 PRICE protocols [22,28,41].

Additionally, ongoing debates surrounding the ideal technique, frequency, and optimal duration of cryotherapy for lateral ligament injuries expose controversies [22,41]. However, the general recommendations suggest that cryotherapy is effective in the first 3 to 5 days after the injury. Consequently, integration of cryotherapy in the initial days of injury through cold whirlpools, compressive cuffs containing a circulating coolant, or ice bags expedites recovery and return to normal activities [41].

Finally, the use and effectiveness of nonsteroidal anti-inflammatory drugs (NSAIDs) use and effectiveness in treating and relieving pain in acutely sprained ankles without negative outcomes have been subject to prospective, randomized, double-blind trials [43-44]. These studies have demonstrated statistically significant differences in the effectiveness of NSAID treatment compared to placebos, which indicate that NSAIDs are more effective in mitigating short-term pain and disability [45]. A review of 18 patients by Ogilvie-Harris et al. [46] revealed an improvement in the effectiveness of pain relief during short-term follow-ups [45]. Furthermore, the review concluded that all the NSAIDs (celecoxib, diflunisal, piroxicam, ibuprofen, or diclofenac) were equally effective, and none was superior [43,45]. Collectively, this body of evidence suggests that patients experience less pain and recover more rapidly when treated with NSAIDs [43-46].

## Conclusions

Despite their high prevalence, there is a distinct lack of well-conducted studies to guide an evidence-based approach to conservative management of lateral ligament ankle injuries. Based on the review of the available evidence, functional therapy with short-term immobilisation of 5-10 days, NSAIDs for pain relief, and cryotherapy is superior to prolonged cast immobilisation as the method of conservatively managing these injuries to produce positive outcomes and prevent the need for surgical intervention. In clinical practice, the Rice protocol (rest, ice, compression, and elevation) is commonly applied for managing acute injuries in the lateral ligament of the ankle. However, protecting the affected ankle is crucial in managing the inflammation and swelling that arise within the initial 24-48 hours post-trauma; a brief period of immobilisation and protection is recommended for the management of said swelling and pain. Nonetheless, early initiation of functional rehabilitation is essential to enhancing positive and optimal outcomes. Ideally, the limited immobilisation phase allows patients to transition through the acute inflammatory stage and heal rapidly with improved outcomes.

Prolonged cast immobilisation should not be the preferred conservative approach for individuals with acute ankle sprains. The existing evidence consistently supports functional treatment as crucial to a reduced recovery period, greater patient satisfaction, and reduced risks of complications such as deep vein thrombosis, muscular atrophy, joint stiffness, and loss of proprioception. It has added benefits, as there is generally less requirement to redevelop the atrophied muscle before returning to activity and for receiving chemical thromboprophylaxis during treatment. An effective rehabilitation program should incorporate a well-structured implementation plan with adequate monitoring to sustain patient recovery and adherence to rehabilitation schedules as per their recovery progress. Ideally, this should involve collaborations with professional physical therapists or qualified athletic trainers throughout each phase of rehabilitation to enhance client compliance and the efficacy of the adopted treatment modality. 
